# Supplementary material for: Automatic correction of performance drift under acquisition shift in medical image classification
Source: Nat Commun. 2023 Oct 19;14:6608. doi: 10.1038/s41467-023-42396-y (PMC10587231; doi:10.1038/s41467-023-42396-y)
Supplement: Supplementary file 1 — Supplementary Information [file 41467_2023_42396_MOESM1_ESM.pdf]

Supplementary material

Supplementary Note 1: Additional results for scenario 1

Here we show that UPA also works for other operating point choices. Specifically, below we repeat our first experiment "deployment to a new site" with another operating point. In this additional experiment, we chose an operating point on the reference domain such that specificity is equal to 90%. Our goal then is to maintain this 90%-specificity operating point on the target domain after applying UPA.

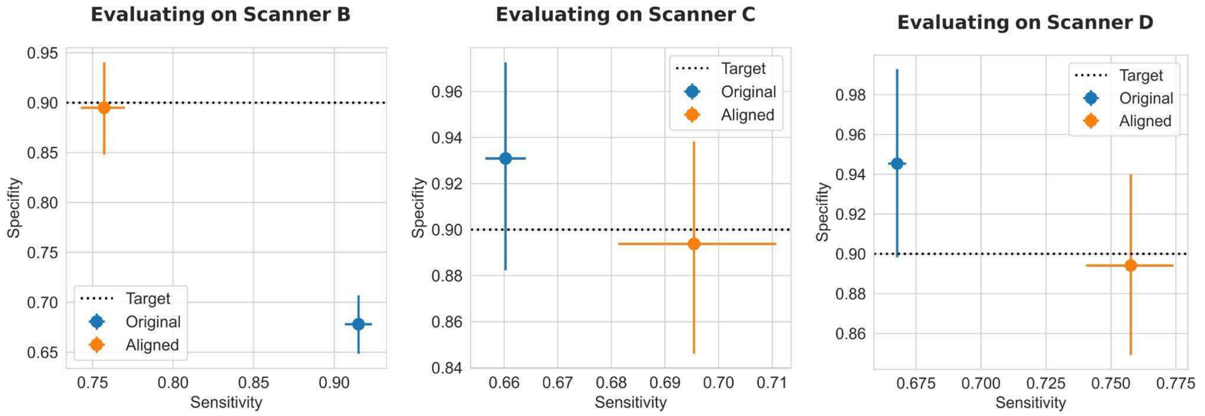

Supplementary Figure 1. Scenario 1: Deployment to a new site - breast screening task – 90%-specificity operating point. Specificity in function of sensitivity before and after prediction alignment. For this analysis, we sample an evaluation set (of 2,500 cases) and a disjoint alignment set (of 1,000 cases) from all available cases, this sampling is repeated 500 times with replacement to yield 500 bootstrap samples. Sensitivity, specificity, ROC-AUC are reported in terms of average results over the bootstrap samples and error bars depict the 95%-bootstrap confidence interval for each metric. UPA is effective at recovering the desired specificity across all out-of-distribution datasets. Source data are provided as a Source Data file.

Supplementary Note 2: Additional results for the effect of the dataset size

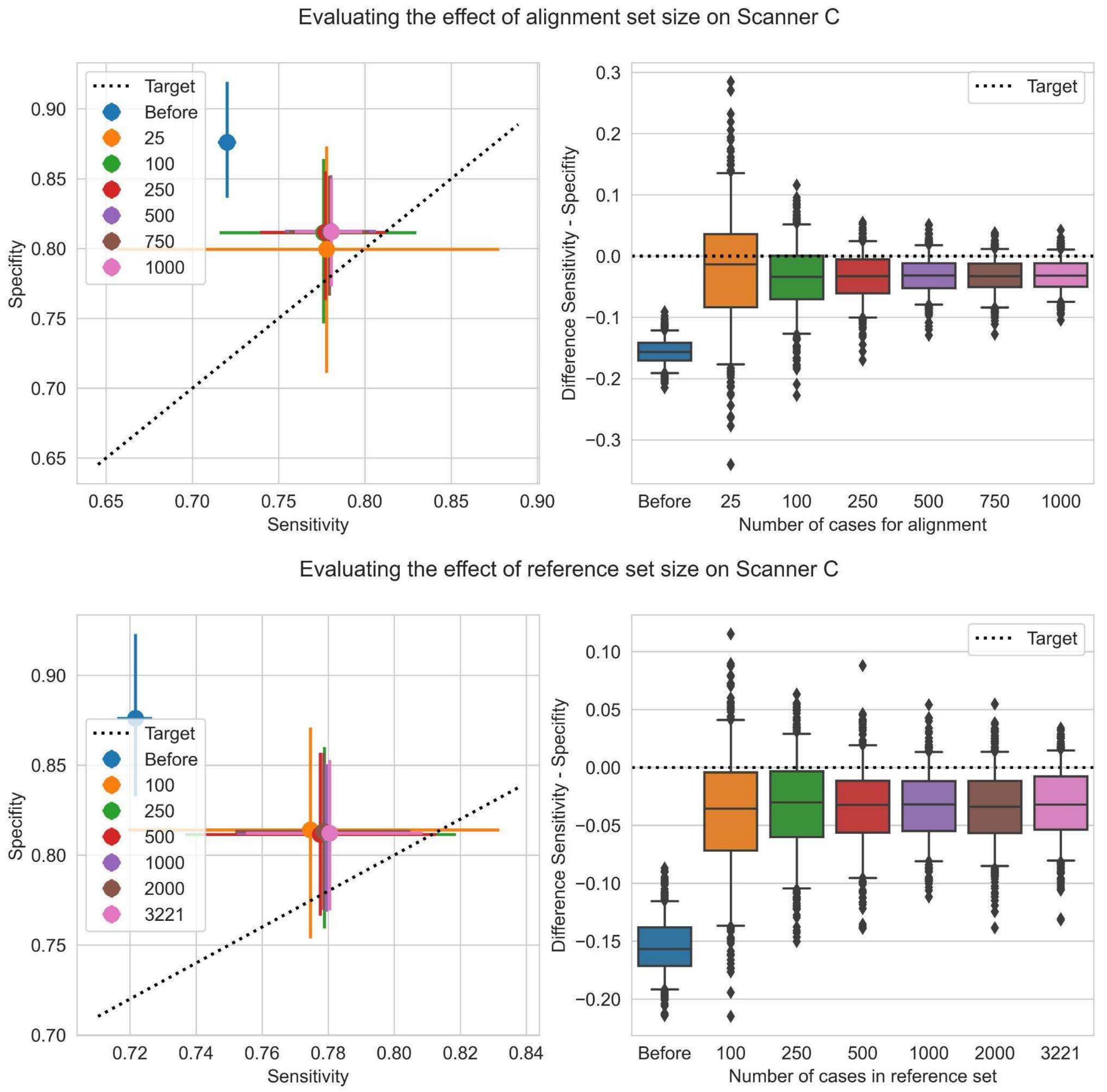

Supplementary Figure 2. Sensitivity analysis for the effect of the dataset size for Scanner C dataset. In the sensitivity analysis on the size of the alignment set (top), we used the full reference set (3,221 cases). Results are reported over 500 bootstrap samples of evaluation sets, alignment sets and reference sets. For the alignment size analysis, each bootstrap sample is created by sampling one alignment set of the size of interest from all available cases as well as one evaluation set (n=2,500 cases). For the reference size analysis, each bootstrap sample is created by sampling one reference set of the size of interest from all available cases as well as one evaluation set (n=2,500 cases). On the left, the points depict the average SPEC / SEN over samples and errors bars represent the 95% bootstrap confidence interval. On the right, each box shows the 25%, 50% and 75% percentiles of the bootstrap distribution; whiskers denote the 5% and 95% percentiles and any point outside of this range is represented as an outlier**.** Source data are provided as a Source Data file.

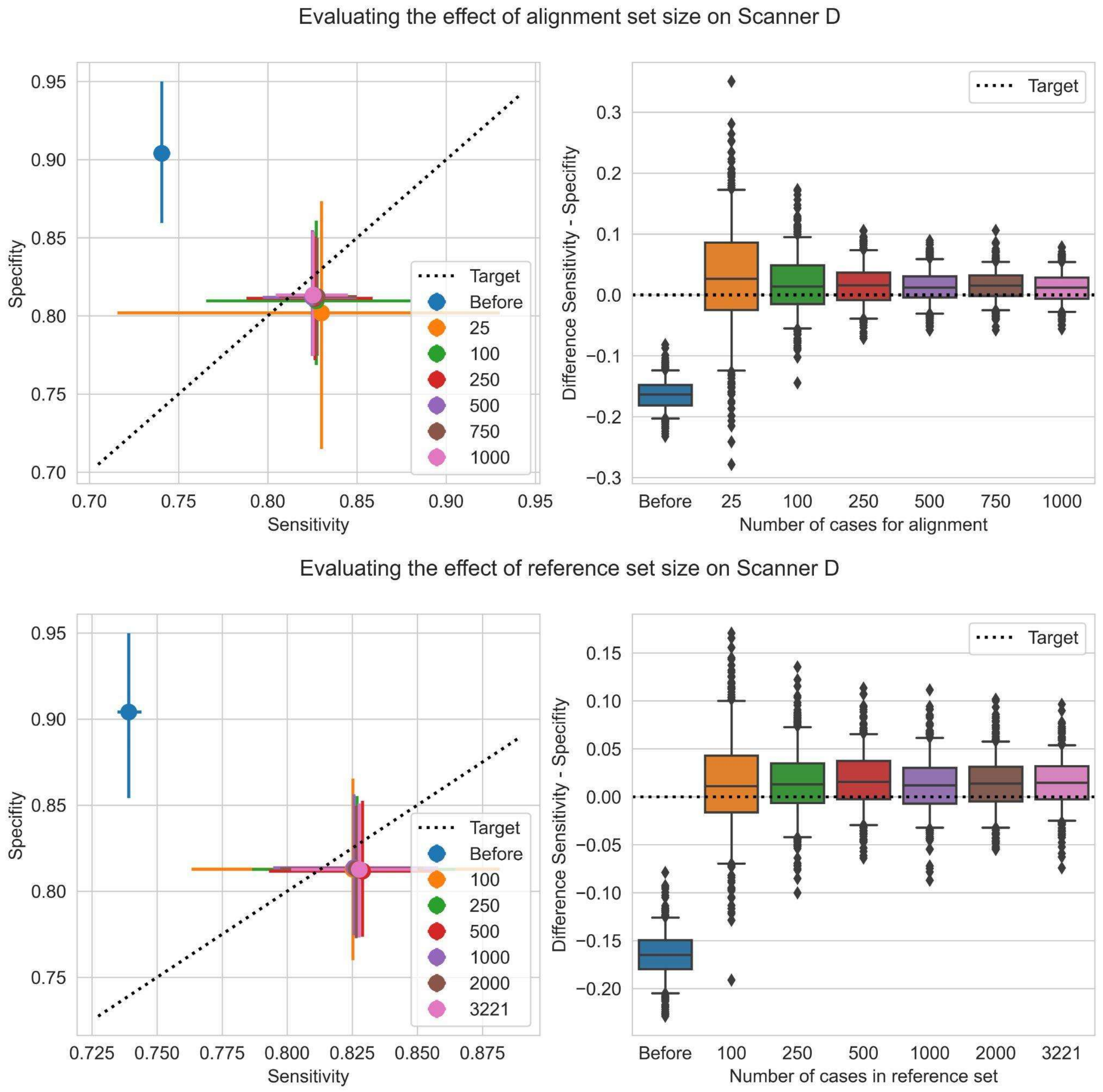

Supplementary Figure 3. Sensitivity analysis for the effect of the dataset size for Scanner D dataset. In the sensitivity analysis on the size of the alignment set (top), we used the full reference set (3,221 cases). Results are reported over 500 bootstrap samples of evaluation sets, alignment sets and reference sets. For the alignment size analysis, each bootstrap sample is created by sampling one alignment set of the size of interest from all available cases as well as one evaluation set (n=2,500 cases). For the reference size analysis, each bootstrap sample is created by sampling one reference set of the size of interest from all available cases as well as one evaluation set (n=2,500 cases). On the left, the points depict the average SPEC / SEN over samples and errors bars represent the 95% bootstrap confidence interval. On the right, each box shows the 25%, 50% and 75% percentiles of the bootstrap distribution; whiskers denote the 5% and 95% percentiles and any point outside of this range is represented as an outlier. Source data are provided as a Source Data file.

Supplementary Note 3: Python pseudo-code for UPA

```
class HistogramMatchEstimator:
    def fit(self, adaptation_set_predictions: np.ndarray, reference_predictions: np.ndarray):
        """
        Fits a linear interpolator to match the cumulative distributions of the reference and adaptation
        set distributions
        """
        # Get both cumulative distribution
        adaptation_quantiles, self.orig_adaptation_values =
self.get_cumulative_density(adaptation_set_predictions)
        ref_quantiles, ref_values = self.get_cumulative_density(reference_predictions)

        # Match both observed cdf by linear interpolation
        self.matched_adaptation_values = np.interp(adaptation_quantiles, ref_quantiles, ref_values)

    def predict(self, test_prediction: np.ndarray):
        """
        Adapts new test prediction by applying the fitted linear interpolator
        """
        return np.interp(test_prediction, self.orig_adaptation_values, self.matched_adaptation_values)

    def get_cumulative_density(self, observations: np.ndarray):
        """
        Returns empirical cumulative distribution function based on array of observations.
        """
        values, counts = np.unique(observations.ravel(), return_counts=True)
        quantiles = np.cumsum(counts) / observations.size
        return quantiles, values
```

Supplementary Note 4: Detection of prevalence shift with UPA

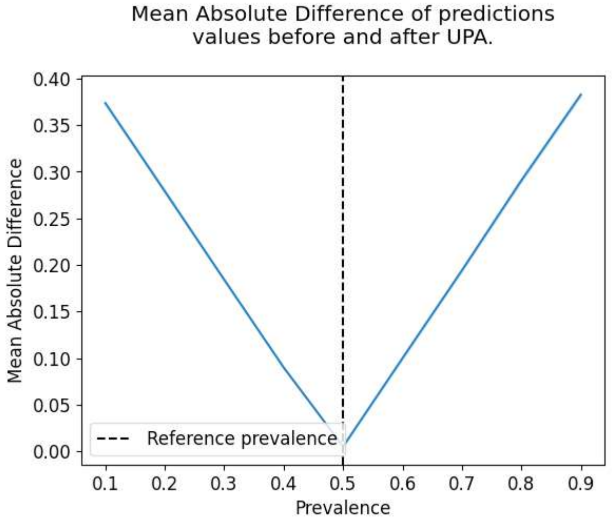

Supplementary Figure 4. Additional experiment illustrating UPA's ability to detect prevalence shift. We simulate the case where there is prevalence shift instead of acquisition shift between the reference and the "deployment" data, using the example of the histopathology task. We sample three disjoint sets from the in-distribution: the reference set (5000 samples), the alignment set (N=5000) and the evaluation set (N=15,000). Here there is no acquisition shift between reference and deployment data (alignment/evaluation set). Instead, we sample alignment and evaluation sets such that they do not exhibit the same prevalence as the reference set (i.e. prevalence shift) and apply UPA to align the predictions. We then plot the Mean Absolute Difference between original and aligned predictions on the evaluation set in function of the prevalence on the shifted data, while keeping the prevalence in the reference set fixed. We plot results over 500 bootstrap samples. There is a clear correlation between the amount of prevalence shift and the observed differences between original and aligned predictions. Source data are provided as a Source Data file.
