# Supplementary material for: Automatic correction of performance drift under acquisition shift in medical image classification
Source: Nat Commun. 2023 Oct 19;14:6608. doi: 10.1038/s41467-023-42396-y (PMC10587231; doi:10.1038/s41467-023-42396-y)
Supplement: Supplementary file 3 — Reporting Summary [file 41467_2023_42396_MOESM3_ESM.pdf]

nature portfolio

Corresponding author(s): Mélanie Roschewitz, Ben Glocker

Last updated by author(s): Sep 5, 2023

Reporting Summary

Nature Portfolio wishes to improve the reproducibility of the work that we publish. This form provides structure for consistency and transparency in reporting. For further information on Nature Portfolio policies, see our Editorial Policies and the Editorial Policy Checklist.

Statistics

For all statistical analyses, confirm that the following items are present in the figure legend, table legend, main text, or Methods section.

n/a	Confirmed	
☐	☒	The exact sample size (*n*) for each experimental group/condition, given as a discrete number and unit of measurement
☐	☒	A statement on whether measurements were taken from distinct samples or whether the same sample was measured repeatedly
☒	☐	The statistical test(s) used AND whether they are one- or two-sided<br>*Only common tests should be described solely by name; describe more complex techniques in the Methods section.*
☒	☐	A description of all covariates tested
☒	☐	A description of any assumptions or corrections, such as tests of normality and adjustment for multiple comparisons
☐	☒	A full description of the statistical parameters including central tendency (e.g. means) or other basic estimates (e.g. regression coefficient) AND variation (e.g. standard deviation) or associated estimates of uncertainty (e.g. confidence intervals)
☒	☐	For null hypothesis testing, the test statistic (e.g. *F*, *t*, *r*) with confidence intervals, effect sizes, degrees of freedom and *P* value noted<br>*Give P values as exact values whenever suitable.*
☒	☐	For Bayesian analysis, information on the choice of priors and Markov chain Monte Carlo settings
☒	☐	For hierarchical and complex designs, identification of the appropriate level for tests and full reporting of outcomes
☒	☐	Estimates of effect sizes (e.g. Cohen's *d*, Pearson's *r*), indicating how they were calculated

Our web collection on statistics for biologists contains articles on many of the points above.

Software and code

Policy information about availability of computer code

Data collection	No software was used for data collection as no data was collected.
Data analysis	Data analysis was performed with Python 3. The implementation of UPA together with code to reproduce all results and figures are made available on https://github.com/biomedia-mira/upa. All system requirements and required packages are listed in the released repository.

For manuscripts utilizing custom algorithms or software that are central to the research but not yet described in published literature, software must be made available to editors and reviewers. We strongly encourage code deposition in a community repository (e.g. GitHub). See the Nature Portfolio guidelines for submitting code & software for further information.

Data

Policy information about availability of data

All manuscripts must include a data availability statement. This statement should provide the following information, where applicable:

- Accession codes, unique identifiers, or web links for publicly available datasets
- A description of any restrictions on data availability
- For clinical datasets or third party data, please ensure that the statement adheres to our policy

All anonymised model outputs supporting the findings described in this manuscript are publicly available in our code repository on https://github.com/biomedia-mira/upa. Source data are provided with this paper which includes the data used to plot the graphs shown in the figures, as well as tables. Access to the OPTIMAM breast cancer dataset can be requested on the project's website: https://medphys.royalsurrey.nhs.uk/omidb/. The WILDS-Camelyon dataset is publicly available under a Creative Commons CC0 license as part of the WILDS benchmark and is readily available for download to anyone. Downloading instructions can be found at

https://wilds.stanford.edu/get_started/. Official data splits were used as part of this study. Raw images from the internal breast cancer imaging datasets from the UK and Hungary were obtained under commercial licences and are not publicly available. Requests for further information can be made via email to the corresponding authors and will be processed within four weeks.

Research involving human participants, their data, or biological material

Policy information about studies with human participants or human data. See also policy information about sex, gender (identity/presentation), and sexual orientation and race, ethnicity and racism.

Reporting on sex and gender	All breast cancer data used was from female participants. Sex and gender information is not made available in the public histopathology dataset.
Reporting on race, ethnicity, or other socially relevant groupings	Information on race, ethnicity or other socially relevant demographics were not available for this study.
Population characteristics	Presence of disease was the primary characteristic used in this study, disease prevalence across different datasets is reported in Table 1 and 2. Additionally, the image acquisition characteristics (e.g. type of scanner, imaging protocol etc.) was used for performance analysis. Other population characteristics such as age were not available and not considered relevant for the algorithmic performance analysis concerning acquisition shift.
Recruitment	No participant were recruited as part of this study.
Ethics oversight	The internal breast cancer datasets from the UK and Hungary were collected previously with ethical approval from the UK National Health Service (NHS) Health Research Authority (HRA) (Reference: 19/HRA/0376) and the Medical Research Council, Scientific and Research Ethics Committee in Hungary (ETT-TUKEB) (Reference: OGYÉI/46651–4/2020). The original study was performed in accordance with the principles outlined in the Declaration of Helsinki for all human experimental investigations. The need for informed consent to participate was reviewed by HRA and ETT-TUKEB and confirmed to not be required as the study involved secondary use of retrospective and pseudonymised data. The present study made secondary use of a fully anonymised version of this previously collected data. For the OPTIMAM breast cancer dataset, the OPTIMAM project obtained renewed HRA approval and a favourable ethical opinion in July 2019 for a renewable period of five years, to collect images and data from participating sites for the creation of a research database and to add new collection sites and was granted a waiver of consent (more information can be found on the project's website: https://medphys.royalsurrey.nhs.uk/omidb/project-information/approval-ethics/). The use of the histopathology datasets is exempt from ethical approval as the analysis is based on fully anonymised, secondary data which is publicly available.

Note that full information on the approval of the study protocol must also be provided in the manuscript.

Field-specific reporting

Please select the one below that is the best fit for your research. If you are not sure, read the appropriate sections before making your selection.

[x] Life sciences [ ] Behavioural & social sciences [ ] Ecological, evolutionary & environmental sciences

For a reference copy of the document with all sections, see nature.com/documents/nr-reporting-summary-flat.pdf

Life sciences study design

All studies must disclose on these points even when the disclosure is negative.

Sample size	Largest possible sets were used from all datasets.
Data exclusions	No further data was excluded from the study sample.
Replication	The experiments have been run over 500 bootstrap samples and no significant differences have been found between individual runs. Confidence intervals are provided for all results.
Randomization	For the histopathology dataset, the official data splits were used. Random sampling was used to create reference and alignment sets, with 500 repetitions to calculate confidence intervals.
Blinding	In this study, we conducted an algorithmic performance analysis using only quantitative and objective statistical measures and not qualitative evaluation was used. There was no risk of bias in the performance comparison and hence no blinding in the data analysis was considered relevant. Additionally, no human intervention or human assessment of medical images was involved.

Reporting for specific materials, systems and methods

We require information from authors about some types of materials, experimental systems and methods used in many studies. Here, indicate whether each material, system or method listed is relevant to your study. If you are not sure if a list item applies to your research, read the appropriate section before selecting a response.

Materials & experimental systems

n/a	Involved in the study
☒	☐ Antibodies
☒	☐ Eukaryotic cell lines
☒	☐ Palaeontology and archaeology
☒	☐ Animals and other organisms
☒	☐ Clinical data
☒	☐ Dual use research of concern
☒	☐ Plants

Methods

n/a	Involved in the study
☒	☐ ChIP-seq
☒	☐ Flow cytometry
☒	☐ MRI-based neuroimaging
