# Supplementary material for: Automatic correction of performance drift under acquisition shift in medical image classification
Source: Nat Commun. 2023 Oct 19;14:6608. doi: 10.1038/s41467-023-42396-y (PMC10587231; doi:10.1038/s41467-023-42396-y)
Supplement: Supplementary file 4 — Description of Additional Supplementary Files [file 41467_2023_42396_MOESM4_ESM.pdf]

File name: Supplementary Code

Description: This repository contains all the necessary code to reproduce the experiments in our manuscript "*Automatic correction of performance drift under acquisition shift in medical image classification*".
